# Hyperhomocysteinemia Concurrent with Metabolic Syndrome Is Independently Associated with Chronic Kidney Disease among Community-Dwelling Adults in an Urban Korean Population

**DOI:** 10.3390/ijerph17186810

**Published:** 2020-09-18

**Authors:** Hana Moon, Hae-Jin Ko, A-Sol Kim

**Affiliations:** 1 Department of Family Medicine, School of Medicine, Daegu Catholic University, Daegu 42472, Korea; tnas1103@naver.com; 2 Department of Family Medicine, School of Medicine, Kyungpook National University, Daegu 41944, Korea; deepai@knu.ac.kr; 3 Department of Family Medicine, Kyungpook National University Hospital, Daegu 41944, Korea; 4 Department of Family Medicine, Kyungpook National University Chilgok Hospital, Daegu 41404, Korea

**Keywords:** metabolic syndrome, homocysteine, chronic kidney disease, proteinuria, cross-sectional study

## Abstract

Elevated homocysteine (Hcy) levels and metabolic syndrome (MetS) are associated with chronic kidney disease (CKD). We investigated the combined effects of hyperhomocysteinemia (HHcy) and MetS on CKD among community-dwelling adults in an urban area of South Korea. We also identified the combination of HHcy and individual MetS components associated with the maximal risk of CKD. A retrospective cross-sectional study involving 19,311 health examinees between 2 January 2011 and 31 December 2015 was conducted. The participants were divided into four groups—namely, the HHcy−/MetS−, HHcy−/MetS+, HHcy+/MetS−, and HHcy+/MetS+ groups. CKD was defined as a low eGFR <60 mL/min/1.73 m^2^ or albuminuria. The HHcy+/MetS+ group had a higher risk of CKD than the HHcy−/MetS+ group (odds ratio (OR): 1.750, *p* = 0.002 for males; OR: 3.224, *p* < 0.001 for females). The HHcy+/MetS+ group had a higher CKD risk than the HHcy+/MetS− group; however, the difference was not statistically significant (OR: 1.070, *p* = 0.712 for males; OR: 1.847, and *p* < 0.074 for females). HHcy concurrent with MetS increased the CKD risk. Among the combinations of HHcy and MetS components, the coexistence of HHcy and central obesity had the greatest effect on CKD. Therefore, the timely detection and treatment of HHcy and MetS are important for preventing CKD.

## 1. Introduction

Chronic kidney disease (CKD) is common worldwide, and several attempts have been made to identify its modifiable risk factors, because the early identification of high-risk patients allows targeted interventions that may prevent CKD progression and improve patient outcomes.

Homocysteine (Hcy) and metabolic syndrome (MetS) are also of great interest to nephrologists because they contribute to the high risk of cardiovascular events in CKD patients. Hcy is considered a marker of vascular disease, particularly in the Asian population, whose daily folate intake is low [1]. Elevated Hcy concentration was considered a consequence of CKD, not a cause of CKD. However, accumulating evidence suggests that hyperhomocysteinemia (HHcy) may be a risk factor for CKD as well. According to a meta-analysis of 41 studies involving 26,617 participants, the Hcy levels significantly and inversely correlated with a decrease in the estimated glomerular filtration rate (eGFR) [2]. A recent prospective study in hypertensive patients reported that HHcy predicts renal function decline [3]. Elevated Hcy concentrations may lead to the development of CKD via various mechanisms, such as oxidative stress and endothelial dysfunction [4]. In contrast, insulin resistance caused by MetS plays a central role in adverse MetS effects on CKD [5]. A meta-analysis of 11 prospective cohort studies involving 30,146 participants reported that the presence of MetS is associated with CKD development [6].

These findings have important clinical implications in South Korea, because CKD is a major health problem in the country, with a high prevalence (7.9%), high mortality rate (4.5%), and associated high medical costs (USD 5 billion in 2011) [7,8,9]. Despite the importance of its early detection, CKD often remains undetected until its later stages. According to a previous study on the South Korean population, only 2% of CKD patients were aware that they had impaired kidney function in the early stage [10]. The prevalence of MetS in this region is as high as 20.3%, and people in South Korea may be at risk of elevated Hcy concentrations as foods in the country are not supplemented with folic acid [11]. The relationship between HHcy and CKD or between MetS and CKD has been previously reported. However, it is not known whether the coexistence of HHcy and MetS has a negative effect on CKD risk. Although previous studies have compared the odds of CKD in association with the components of MetS, it is also important to assess if the combination of HHcy and individual MetS components has a maximal effect on the CKD risk, because this may aid in the identification of high-risk individuals and guide targeted prevention and lifestyle modification strategies. Therefore, we aimed to: (1) evaluate whether the coexistence of HHcy and MetS increases the CKD risk among community-dwelling adults in urban southeast Korea, and (2) identify the combination of HHcy and individual MetS components that maximally increases the CKD risk (i.e., HHcy+/Central obesity+, HHcy+/High triglycerides (TG)+, HHcy+/Low high-density lipoprotein-cholesterol (HDL)+, HHcy+/High blood pressure (BP)+, HHcy+/High fasting glucose+).

## 2. Materials and Methods

### 2.1. Subjects

This study involved 23,352 community-dwelling adults who underwent annual health examinations at two health promotion centers in Daegu city, South Korea, from 2 January 2011 to 31 December 2015. The inclusion criteria were as follows: those ≥ 19 years of age or older, residents of Daegu, and participants who were Korean. The exclusion criteria were as follows: age < 19 years (*n* = 72), incomplete data (*n* = 3217), menstruation on the examination day (*n* = 87), pregnancy (*n* = 2), and pyuria and suspected urinary tract infection (*n* = 28). In addition, patients assigned the International Statistical Classification of Diseases and Related Health Problems (ICD) codes for a current or past diagnosis of cancer (*n* = 173), liver disease (*n* = 162), ischemic heart disease (*n* = 149), pulmonary disease (*n* = 133), organ transplantation (*n* = 2), and kidney disease (*n* = 16) were excluded (Table S1). Finally, 19,311 participants were included in our analysis.

This study was in accordance with the Helsinki Declaration and was approved by the institutional review board (IRB, protocol number: KNUH 2015-02-021). The requirement for informed consent was waived by the IRB due to the retrospective design of the study.

### 2.2. Assessment of Body Characteristics and Health-Related Behaviors

Body characteristics and health-related behaviors were retrospectively retrieved from the hospital database for analysis. Data collection included height, weight, waist circumference, blood pressure, self-reported alcohol intake, smoking status, and physical activity. Body mass index (BMI) was calculated by dividing the weight in kilograms by the square of the height in meters (kg/m^2^).

### 2.3. Biochemical Assessments

Laboratory test results were retrospectively retrieved for analysis. Overnight fasting blood and mid-stream urine samples were collected. For measuring the serum Hcy levels, fresh venous samples were kept on crushed ice and centrifuged within an hour. The serum collected from each participant was isolated by centrifugation (F5220, Kubota Corporation, Saitama, Japan) and kept refrigerated (2–8 °C) for testing within 48 h. The total serum L-homocysteine concentrations were measured using fluorescent polarization immunoassay kits (DZ568A-K homocysteine enzymatic assay kit, Diazyme Laboratories, CA, USA) on a Modular P 800 analyzer (Roche Diagnostics, Mannheim, Germany). The intra- and inter-assay coefficients of variation were both <5%. The measuring range of the assay was from 3 to 50 μmol/L.

Other biochemical parameters, including total cholesterol (TC), low-density lipoprotein-cholesterol (LDL), high-density lipoprotein-cholesterol (HDL), triglycerides (TG), high-sensitivity c-reactive protein (hs-CRP), albumin, uric acid, fasting plasma glucose, and hemoglobin A1c, were analyzed through standard clinical chemistry laboratory techniques using a D-2400 Hitachi analyzer (Hitachi High-Technologies Corporation, Tokyo, Japan). The serum creatinine concentrations were assessed using Jaffe’s method on an AU 2700 Beckman Coulter analyzer (Beckman Coulter Diagnostics, La Brea, CA, USA). The creatinine calibration was traceable to an isotope dilution mass spectrometry.

The results of urine dipsticks (Uriscan 10 SGL strip, YD-Diagnostics, Yongin, South Korea) for albuminuria were categorized as normal to mildly increased (A1) (negative to trace), moderately increased (A2) (trace to 1+ positive), or severely increased (A3) (2+ or greater) [12].

eGFR was calculated using the 2009 CKD Epidemiology Collaboration creatinine equation: 141 × min(SCr/𝜿, 1)^α^ × max(SCr/𝜿, 1)^−1.209^ × 0.993^Age^ [× 1.018 if female] [× 1.159 if black]. Here, SCr is serum creatinine (in mg/dl), 𝜿 is 0.7 for females and 0.9 for males, α is −0.329 for females and −0.411 for males, min is the minimum of SCr/𝜿 or 1, and max is the maximum of SCr/𝜿 or 1 [12].

### 2.4. Definition of Term

HHcy was defined as a serum Hcy concentration >15 μmol/L [13]. MetS was diagnosed according to the Modified National Cholesterol Education Program Adult Treatment Panel III (NCEP-ATP III) guidelines and according to the waist circumference values for Koreans suggested by the Korean Society for the Study of Obesity [14,15]. At least three or more of the following five components are needed to diagnose MetS: central obesity, high TG, low HDL, high blood pressure (≥130/85 mmHg or the use of antihypertensive medication), and high fasting glucose (≥100 mg/dL or the use of anti-diabetic medication). Central obesity was defined as a waist circumference of ≥90 cm for males and ≥85 cm for females. High TG was defined as TG ≥ 150 mg/dL, and low HDL was defined as HDL cholesterol <40 mg/dL for males and <50 mg/dL for females. CKD was defined as the presence of low eGFR or albuminuria [12]. Low eGFR was defined as an eGFR < 60 mL/min/1.73 m^2^. Albuminuria was defined as A2 or A3 on urine dipstick tests [12].

### 2.5. Statistical Analyses

All the analyses were conducted using the R statistical package version 3.6.3 (R Foundation for Statistical Computing, Vienna, Austria). The analyzed values were statistically significant when a value of *p* < 0.05 was obtained. The participants were divided into four groups according to their HHcy and MetS status: HHcy−/MetS−, HHcy−/MetS+, HHcy+/MetS−, HHcy+/MetS+. As shown in Table 1, the normality of data was assessed using a Shapiro–Wilk test. Continuous variables were compared by an analysis of variance, whereas categorical variables were compared by Pearson’s chi-square tests. Bonferroni post-hoc correction was used for multiple comparisons. Continuous variables were presented as means ± standard deviations and categorical variables as numbers (percentages).

Because the serum Hcy concentrations and prevalence of MetS are sex-dependent, males and females were analyzed separately [16,17]. Although the interaction between eGFR and albuminuria is biologically plausible, a meta-analysis indicated that albuminuria and low eGFR might be independent [18]. Therefore, logistic regression analyses were conducted separately for the three dependent variables (CKD, low eGFR, and albuminuria). Multiple logistic regression models were applied to calculate the odds ratios for having CKD, low eGFR, and albuminuria associated with the presence of HHcy, MetS, and the individual components of MetS. First, logistic regression analyses were used to examine the combined effects of HHcy and MetS on CKD, low eGFR, and albuminuria. The HHcy−/MetS− group was considered as the reference group. Second, logistic regression analyses were used to examine the association between individual components of MetS and CKD, low eGFR, and albuminuria. Third, logistic regression analyses were used to examine the odds of CKD, low eGFR, and albuminuria, and their association with the combination of HHcy and the components of MetS.

The following models were run in each logistic regression analysis: Model 1 was adjusted for age only. Model 2 was adjusted for age, cigarette smoking and drinking status, physical activity level, hs-CRP level, and albumin level, because these have been previously identified as possible factors that influence the parameters of renal function [19,20,21]. The *p*-for trend was calculated using the Cochran–Armitage trend test. Additionally, conditional logistic regression after propensity score-matching in MetS patient subgroups was conducted. Propensity scores were calculated with a logistic regression analysis, which included sex, age, cigarette smoking, drinking status, physical activity level, hs-CRP level, and albumin level as covariates. The patients were matched at a 1:1 ratio by the nearest-neighbor method.

## 3. Results

### 3.1. Characteristics of the Study Population

The total study population consisted of 19,311 Korean adults, including 10,843 (56.2%) males and 8468 (43.9%) females. Overall, 1730 (9.0%) had HHcy and 4091 (21.2%) had MetS. The most prevalent component of MetS was hypertension (36.8%), followed by high fasting blood glucose (29.0%), high TG (28.8%), central obesity (23.8%), and low HDL (21.9%). The mean Hcy concentrations were 11.5 ± 4.0 μmol/L for males and 8.4 ± 3.0 μmol/L for females. Males had a higher prevalence of HHcy (13.4% vs. 3.3%) and a lower prevalence of MetS (16.5% vs. 27.2%). Central obesity and low HDL were more prevalent in females than in males (32.7% vs. 16.8%, 35.7% vs. 11.1, respectively). The average eGFR was 96.24 ± 23.39 mL/min/1.73 m^2^ in males and 105.22 ± 22.59 mL/min/1.73 m^2^ in females. Overall, 2.1% of males (229/10,843) had a low eGFR and 11.03% of males (1196/10,843) had albuminuria; 1.3% of females (107/8468) had a low eGFR and 7.5% of females (631/8468) had albuminuria. Sixty-nine males and 28 females had both a low eGFR and albuminuria.

**Figure 1:**
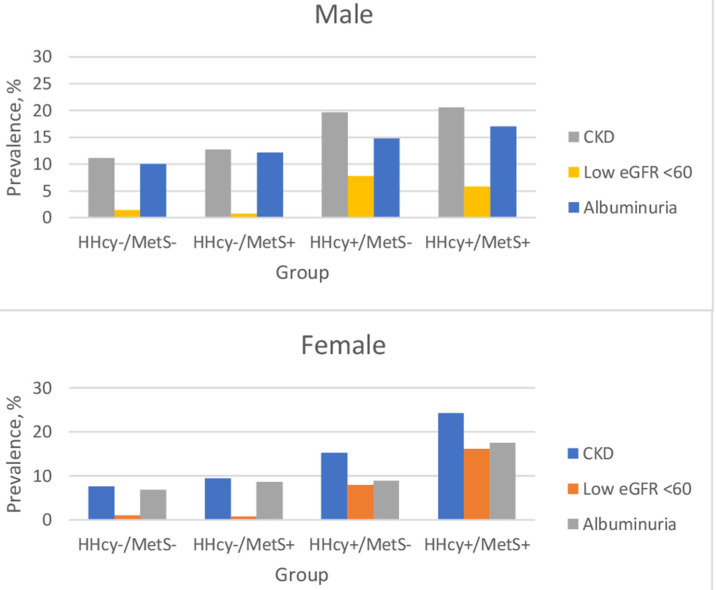
Prevalence of CKD among the four groups. Abbreviations: HHcy, hyperhomocysteinemia; MetS, metabolic syndrome; CKD, chronic kidney disease; eGFR, estimated glomerular filtration rate.

Table 1 displays the characteristics of the study population according to sex and the four groups. Participants with HHcy had a higher prevalence of binge drinking, regardless of the presence of MetS. The participants in the two MetS groups (HHcy−/MetS+, HHcy+/MetS+) had a higher proportion of elevated uric acid concentration than those in the other groups. The prevalence of CKD, low eGFR, and albuminuria in the four groups is shown in Figure 1. The HHcy+/MetS+ group had the highest prevalence of CKD for both sexes (females: 24.3%, vs. 7.6%, *p* < 0.001; males: 20.6% vs. 11.1%, *p* < 0.001), followed by the HHcy+/MetS−, HHcy−/MetS+, and HHcy−/MetS− groups.

### 3.2. Association between the Presence of HHcy and MetS, and CKD

The multivariable-adjusted odds ratios (ORs) and 95% CIs for CKD among the four groups are presented in Table 2. The coexistence of HHcy and MetS had the greatest effect on CKD development. In Model 2, the participants in the HHcy+/MetS+ group were twice to four times more likely to have CKD compared with those in the reference group (OR: 2.015, *p* < 0.001 for males; OR: 4.006, *p* < 0.001 for females). The HHcy+/MetS+ group had a higher CKD OR than the HHcy−/MetS+ group (OR: 1.750, *p* = 0.002 for males; OR: 3.224, *p* < 0.001 for males). The HHcy+/MetS+ group had a higher CKD risk than the HHcy+/MetS− group, though the risk was not statistically significant (OR: 1.070, *p* = 0.712 for males; OR: 1.847, *p* < 0.074 for females).

### 3.3. Association between the Presence of HHcy and MetS, and Low eGFR

Separate logistic regression analyses were conducted for low eGFR and albuminuria, respectively. The multivariable-adjusted ORs and 95% CIs for low eGFRs among the four groups are presented in Table 3. In Model 2, in females, participants in the HHcy+/MetS+ group were 13.5 times more likely to have a low eGFR compared with those in the reference group (OR: 13.530, *p* < 0.001). However, in males, the OR for low eGFR for the HHcy−/MetS+ group compared to that for the reference group was 0.540 (*p* = 0.046). In males, the OR for low eGFR for the HHcy+/MetS+ group compared to the HHcy+/MetS− group was 0.787, though the difference was not statistically significant (*p* = 0.460).

### 3.4. Association between the Presence of HHcy and MetS, and Albuminuria

The multivariable-adjusted ORs and 95% Cis for albuminuria among the four groups are presented in Table 4. The coexistence of Hhcy and MetS had the greatest effect on albuminuria development. In Model 2, participants in the Hhcy+/MetS+ group were 1.7 to three times more likely to have albuminuria compared with those in the reference group (OR: 1.769, *p* = 0.002 for males; OR: 3.186, *p* < 0.001 for females). The OR of albuminuria for the Hhcy+/MetS+ group compared to that for the Hhcy−/MetS+ group 2.534 (*p* = 0.004), and the OR for albuminuria for the Hhcy+/MetS+ group compared to the Hhcy+/MetS− group was 2.241 (*p* = 0.044) for females.

We further conducted a subgroup analysis in the MetS patients. As shown in Table S2, the CKD OR for the Hhcy+ group compared to that for the Hhcy− group was 1.750 (*p* = 0.002) for males, and 3.224 (*p* < 0.001) for females. In addition, we conducted a propensity score-matched analysis in a subgroup of MetS patients (Table S3). Two hundred and ninety-seven patients in the HHcy+ group were matched with 297 patients in the HHcy− group using the nearest-neighbor matching method. The eGFR OR for the HHcy+ group compared to that for the HHcy− group was 6.692 (*p* < 0.0001).

### 3.5. Odds of CKD, Low eGFR, and Albuminuria for Individual MetS Components

We further examined the associations of individual components of MetS with the risk of CKD, low eGFR, and albuminuria. As shown in Table S4, among five components, only the presence of central obesity was significantly associated with CKD in both sexes (OR: 1.228, *p* = 0.006 for males, OR: 1.401, *p* < 0.001 for females). None of the components of MetS had a statistically significant association with low eGFR in both sexes. Only the presence of central obesity was significantly associated with albuminuria in both sexes (OR: 1.308, *p* < 0.001 for males, OR: 1.415, *p* < 0.001 for females). High TG was also found to be associated with albuminuria in males (OR: 1.164, *p* = 0.022). A low HDL and high blood pressure were also risk factors for albuminuria in females (OR: 1.270, *p* = 0.005; OR: 1.210, *p* = 0.025).

### 3.6. Odds of CKD, Low eGFR, and Albuminuria for the Combination of HHcy and MetS Components

The odds of CKD, low eGFR, and albuminuria for the combination of HHcy and MetS components are shown in Tables S5–S7. The coexistence of HHcy and central obesity had the greatest effect on the development of CKD, low eGFR, and albuminuria. The HHcy+/Central obesity+ group had a higher CKD risk than of the HHcy−/Central obesity+ group in females (OR: 1.460, *p* = 0.033), though this risk was not significantly different in males. The HHcy+/Central obesity+ group also had a higher CKD risk than the HHcy+/Central obesity− group in females (OR: 2.322, *p* = 0.012), though not for males.

## 4. Discussion

To the best of our knowledge, this is the first study to suggest that the coexistence of HHcy and MetS increases the risk of CKD, specifically low eGFR and albuminuria, among community-dwelling adults in an urban area. We also found that HHcy or MetS alone may increase CKD risk, aligning with previous studies [2,3,22], and this finding supports the role of HHcy as a contributor to CKD. This study also identified the MetS component that maximally increases the risk of CKD. Among the combinations of HHcy and MetS components, the coexistence of HHcy and central obesity had the greatest effect on CKD. In this study, central obesity (in both sexes), high TG (male), low HDL levels (female), and high blood pressure (female) were the MetS components associated with albuminuria. However, our findings were not consistent with previous studies. Jiang et al. reported that high blood pressure and high fasting blood glucose, but neither high TG nor low HDL were associated with an increased risk for CKD [23]. Another study with a four-year follow-up of 4326 participants reported that high TG in females and low HDL in males were correlated with a decline in eGFR and that high TG in both males and females was predictive of increased proteinuria [24]. Zeng et al. showed that people with elevated TG and central obesity were more likely to have CKD than those with normal TG and normal waist circumference (OR 1.95, 95% CI 1.32–2.88, *p* = 0.001) [25]. A Chinese population-based cross-sectional study on serum lipids and CKD showed that only high TG was an independent predictor for CKD in men and that none of the lipids were predictive in women [26].

A mechanism that might explain the combined effect of HHcy and MetS remains to be elucidated. A possible explanation is that an elevated Hcy concentration can promote CKD, and also indirectly contribute to CKD via MetS. First, HHcy contributes to the development of CKD as a pathogenic factor. Recent studies have revealed the mechanisms of Hcy-induced renal injury, including oxidative stress, inflammation, endothelial dysfunction, and hypomethylation [4]. Oxidative stress is a state of imbalance between free radical production and clearance. Auto-oxidation of Hcy can generate potent reactive oxygen species. When reactive oxygen species are made in excess, they can react with lipids, proteins, and DNA, resulting in cellular damage [27]. Inflammation can accelerate renal dysfunction progression, and Hcy-induced hypomethylation can lead to endothelial cell injury [4]. Another important pathway is via adenosine, which is a constrictor of the renal vascular bed. An elevation of Hcy concentration can decrease adenosine formation, resulting in decreased nitric oxide bioavailability, which contributes to a poor prognosis of CKD [4].

Second, the impact of MetS on CKD is multifactorial. Central obesity and insulin resistance are the most widely accepted key factors implicated in the development of MetS. White adipose tissue is considered as an active endocrine and metabolic organ to produce inflammatory cytokines [28]. Expansion of adipose tissue promotes chronic inflammation and oxidative stress, which exacerbate insulin resistance [28]. These can cause endothelial dysfunction, renin-angiotensin-aldosterone activation. MetS can also lead to mitochondrial dysfunction, which can promote kidney damage. Insulin resistance and chronic inflammation contribute to microvascular remodeling [5]. Insulin resistance may alter renal hemodynamics via activation of the sympathetic nervous system, sodium retention, and downregulation of the natriuretic peptide system [29].

In this regard, high levels of Hcy levels also can partially contribute to the development of MetS, and it may indirectly contribute to CKD. For example, Hcy-mediated DNA hypermethylation leads to abnormal silencing of the genes essential for normal fatty acid metabolism [30]. Under the condition of insulin resistance owing to increased homocysteine concentrations, cells in adipose tissues fail to respond to insulin effectively, which leads to disturbances in lipid metabolism [29]. However, specific pathways to explain the interactions among HHcy, MetS, and kidney dysfunction remain to be explored.

Interestingly, in this study, the group influence on CKD was greater in females than in males as follows: First, for females, the OR for risk of low eGFR in the HHcy+/MetS+ group was almost 3.6 times greater than that for males (13.530 vs. 3.716, respectively). Second, the HHcy+/MetS+ group also showed a higher OR for albuminuria in females than in males (3.186 vs. 1.769, respectively). These differences between sexes were also found in a previous study. In a study conducted by Chen et al., the Hcy level was negatively associated with eGFR, and the association was more profound for females [31]. This may be due to sex differences in body composition. Women have more fat in the abdominal region compared to men with the same BMI [32]. Central obesity may alter renal hemodynamics. Intra-abdominal pressure is increased in people with central obesity, and it causes efferent arteriolar vasoconstriction, leading to glomerular hyperfiltration [33,34]. Here, the central pattern of fat distribution is considered to be a risk factor for glomerular hyperfiltration [23]. Central body fat distribution is also known to be related to a higher risk of microalbuminuria, and it may be explained by leptin secreted from adipocytes [35]. Leptin stimulates renal transforming growth factor beta-1 and increases the glomerular expression of type IV collagen, suggesting a potential mechanism linking abdominal fat to elevated albuminuria [36]. Additionally, in men the presence of MetS alone was associated with a higher eGFR compared to that in the controls. This may be because of the hyperfiltration observed in the initial phases of MetS [37].

The prevalence of CKD in this study was slightly higher than that found in a previous investigation conducted in South Korea in 2011 and 2013 [7]. However, the prevalence of MetS was comparable to previous results reported in a study in 2018 [11]. The prevalence of HHcy was 13.4% for males and 3.3% in females, and these findings were comparable to those from previous reports on the Korean population [38]. The incidence of HHcy varies around the world, with the lowest prevalence in the United States (5%), 6% in Costa Rica, 27.5% in China, and the highest prevalence in India (77%) [39,40,41]. As shown in Table 1, two of the HHcy groups had a higher prevalence of binge drinking, regardless of the presence of MetS. According to Kamat et al. chronic alcohol intake may increase Hcy concentration [42]. In a study by Yu et al. the prevalence of HHcy was higher than that in the overall pooled prevalence in China (39.7% vs. 27.5%) [39]. The study reported that higher alcohol consumption and poorer dietary habits might explain this high prevalence.

This study had several limitations. First, it included only Korean participants; therefore, its results may not apply to other ethnic groups. Second, albuminuria was assessed semi-quantitatively, using a protein dipstick and random urine samples, as this method is the only screening tool used for adults at these health-promotion centers. Therefore, we were unable to use more accurate methods, such as 24-h urine analyses or measurements of the ratio of urinary albumin to creatinine levels (UACR). Third, the influence of vitamins B6 and B12, folic acid, and medications related to kidney function or MetS cannot be ruled out. Fourth, we could not determine a cause–effect relationship because of this study’s cross-sectional design. The nature of the combined effect of HHcy and MetS needs to be clarified. Longitudinal studies should be designed to answer this question.

This study has a number of strengths. First, we lowered the impact of eGFR on the concentration of serum homocysteine. We excluded pre-diagnosed CKD patients, and the included participants had relatively high eGFR levels (96.24 ± 23.39 mL/min/1.73 m^2^ in males; 105.22 ± 22.59 mL/min/1.73 m^2^ in females). Therefore, eGFR is seemingly unlikely to affect serum homocysteine levels in this study. Second, we used methods that are easily applicable in clinical practice. The variables (Hcy, the five MetS components, eGFR, and dipstick urinalysis) used in this study are commonly measured in outpatient clinics or during regular check-ups in South Korea. Although UACR is more accurate in identifying albuminuria, in a primary clinic UACR is rare and dipstick urinalysis/spot urine testing is commonly used. Primary care physicians are the first to respond to cases of kidney dysfunction found incidentally. Therefore, this study provides useful clinical information for clinical practitioners to help them identify high-risk patients and make informed clinical decisions without having specialized equipment at their clinic.

Although managing CKD was traditionally the responsibility of nephrologists, the need for primary care involvement has grown. Early detection and management are necessary to reduce CKD complications and slow its progression to end-stage renal disease. Primary care physicians are best positioned to monitor kidney function regularly and provide lifestyle modification strategies. The fundamentals of healthy living, such as exercise, diet, smoking, and alcohol, may be reiterated to patients, along with strategies for managing the five MetS components. Considering that many CKD patients have multiple comorbidities, and that CKD itself and other chronic disease entities share several pathogenetic mechanisms and risk factors, primary care physicians have an essential role in coordinating their management plan. Importantly, considering that HHcy and MetS are risk factors not only for CKD, but also for cardiovascular disease, detecting individuals who have both HHcy and MetS may help effectively reduce the future burden of these diseases. South Koreans may check all MetS components every two years for free, because the National Health Insurance Service fully covers these tests. Therefore, physicians who thoroughly review the findings of previous check-ups may be able to identify individuals at risk of CKD progression. Those individuals should be the primary targets of lifestyle modification intervention. In particular, people with central obesity and low HDL are often overlooked in clinical practice because they do not present with troublesome symptoms, unlike hypertension, and there is currently no specific medication for these conditions. CKD screening has been recommended in high-risk patients with hypertension or diabetes, though mass screening in the general population is not cost-effective [43]. However, our findings indicate that the HHcy+/MetS+ and HHcy+/Central obesity+ groups should also be the target population of lifestyle intervention in order to delay or prevent CKD development. Early CKD detection is the best prevention. The identification and management of these patient groups should be stressed as a strategy to prevent CKD.

## 5. Conclusions

In this study, the combination of HHcy and MetS increased CKD risk, low eGFR, and albuminuria among community-dwelling Korean adults in an urban area, regardless of age, cigarette smoking, drinking status, physical activity level, HbA1c level, hs-CRP level, and albumin level. Central obesity (in both sexes), high TG (male), low HDL levels (female), and high blood pressure (female) were the MetS components associated with albuminuria. Among the combinations of HHcy and MetS components, the coexistence of HHcy and central obesity had the greatest effect on CKD, low eGFR, and albuminuria. Therefore, the detection and treatment of HHcy and MetS should be stressed as a modifiable target for preventing CKD.

## Figures and Tables

**Table 1:** Characteristics of the study population according to sex and the four groups.

	Male						Female					
	HHcy−/MetS−	HHcy−/MetS+	HHcy+/MetS−	HHcy+/MetS+	Total	*p*	HHcy−/MetS−	HHcy−/MetS+	HHcy+/MetS−	HHcy+/MetS+	Total	*p*
Number of participants	7828 (72.19)	1561 (14.40)	1231 (11.35)	223 (2.06)	10,843		5959 (70.37)	2233 (26.37)	202 (2.39)	74 (0.87)	8468	
eGFR, mL/min/1.73 m^2^	97.34 ± 19.36	96.59 ± 17.73	90.27 ± 43.66	88.10 ± 20.32	96.24 ± 23.39	<0.001	105.96 ± 22.68	105.21 ± 21.10	90.28 ± 25.05	86.97 ± 29.32	105.22 ± 22.59	<0.001
Homocysteine, μmol/L	10.38 ± 2.31	10.15 ± 2.32	19.24 ± 4.53	18.85 ± 4.03	11.52 ± 4.05	<0.001	8.06 ± 2.27	7.97 ± 2.26	18.80 ± 4.35	19.22 ± 3.65	8.39 ± 3.04	<0.001
Age, years	47.86 ± 11.01	47.48 ± 11.40	49.21 ± 12.88	47.65 ± 13.09	47.95 ± 11.35	<0.001	48.07 ± 11.76	47.51 ± 11.77	53.51 ± 15.42	52.61 ± 14.52	48.09 ± 1.93	<0.001
Central obesity	655 (8.37)	937 (60.03)	104 (8.45)	130 (58.30)	1826 (16.84)	<0.001	998 (16.75)	1679 (75.19)	39 (19.31)	56 (75.68)	2772 (32.74)	<0.001
High TG	1460 (18.65)	1276 (81.74)	219 (17.79)	180 (80.72)	3135 (28.91)	<0.001	752 (12.62)	1609 (72.06)	29 (14.36)	54 (72.97)	2444 (28.86)	<0.001
Low HDL	435 (5.56)	633 (40.55)	52 (4.22)	84 (37.67)	1204 (11.10)	<0.001	1230 (20.64)	1699 (76.09)	40 (19.80)	56 (75.68)	3025 (35.72)	<0.001
High blood pressure	2192 (28.00)	1219 (78.09)	353 (28.68)	174 (78.03)	3938 (36.32)	<0.001	1374 (23.06)	1550 (69.41)	55 (27.23)	51 (68.92)	3030 (35.78)	<0.001
High fasting glucose	1655 (21.14)	1190 (76.23)	244 (19.82)	172 (77.13)	3261 (30.07)	<0.001	998 (16.75)	1387 (62.11)	39 (19.31)	35 (47.30)	2459 (29.04)	<0.001
Current smoker	3652 (46.65)	738 (47.28)	565 (45.90)	113 (50.67)	5068 (46.74)	0.898	445 (7.47)	173 (7.75)	12 (5.94)	4 (5.41)	634 (7.49)	0.673
Binge drinker	991 (12.66)	199 (12.75)	168 (13.65)	33 (14.80)	1391 (12.83)	0.331	766 (12.85)	301 (13.48)	29 (14.36)	13 (17.57)	1109 (13.10)	0.797
Physically inactive	3829 (48.91)	751 (48.11)	601 (48.82)	114 (51.12)	5295 (48.83)	0.846	2732 (45.85)	978 (43.80)	98 (48.51)	32 (43.24)	3840 (45.35)	0.294
Hs-CRP, mg/L	0.13 ± 0.44	0.22 ± 0.75	0.12 ± 0.34	0.19 ± 0.48	0.14 ± 0.49	<0.001	0.13 ± 0.63	0.21 ± 1.01	0.11 ± 0.53	0.18 ± 0.42	0.15 ± 0.75	<0.001
Albumin, g/L	4.51 ± 0.29	4.58 ± 0.30	4.53 ± 0.28	4.59 ± 0.28	4.53 ± 0.29	<0.001	4.49 ± 0.29	4.55 ± 0.29	4.55 ± 0.27	4.55 ± 0.26	4.51 ± 0.29	<0.001
Uric acid, mg/dL	5.07 ± 1.37	6.00 ± 1.49	5.05 ± 1.31	6.02 ± 1.45	5.22 ± 1.42	<0.001	4.83 ± 1.38	5.82 ± 1.53	4.93 ± 1.30	6.03 ± 1.27	5.11 ± 1.49	<0.001
WC, cm	78.55 ± 8.60	89.95 ± 7.42	78.63 ± 8.83	90.01 ± 7.15	80.43 ± 9.44	<0.001	77.07 ± 8.36	88.21 ± 7.08	78.07 ± 8.12	87.66 ± 6.13	80.13 ± 9.42	<0.001
TC, mmol/L	195.51 ± 35.44	205.44 ± 40.76	196.85 ± 35.66	206.33 ± 41.05	197.32 ± 36.58	<0.001	194.59 ± 35.49	203.96 ± 39.16	194.44 ± 34.51	213.28 ± 36.04	197.22 ± 36.73	<0.001
TG, mmol/L	114.26 ± 67.60	230.17 ± 133.8	113.61 ± 64.86	207.58 ± 96.84	132.79 ± 91.18	<0.001	104.45 ± 54.40	208.01 ± 123.67	104.43 ± 54.51	216.2 ± 140.61	132.74 ± 92.15	<0.001
LDL, mmol/L	121.21 ± 32.14	127.15 ± 36.56	121.57 ± 32.62	129.93 ± 35.61	122.28 ± 33.02	<0.001	119.67 ± 32.47	127.89 ± 34.12	119.25 ± 32.84	135.89 ± 32.70	121.97 ± 33.14	<0.001
HDL, mmol/L	58.17 ± 14.33	44.98 ± 11.56	58.80 ± 14.14	46.37 ± 12.74	56.10 ± 14.73	<0.001	60.30 ± 14.21	45.70 ± 10.32	59.60 ± 13.03	45.95 ± 11.48	56.31 ± 14.74	<0.001
SBP, mmHg	120.75 ± 14.97	135.29 ± 14.16	121.07 ± 15.37	135.83 ± 15.40	123.19 ± 15.86	<0.001	119.04 ± 14.73	133.60 ± 14.71	120.18± 15.00	132.47± 13.41	123.02 ± 16.07	<0.001
DBP, mmHg	73.68 ± 10.63	83.55 ± 10.48	73.92 ± 10.93	83.47 ± 10.58	75.33 ± 11.25	<0.001	72.09 ± 10.32	82.22 ± 10.53	72.76 ± 10.61	81.70 ± 10.49	74.86 ± 11.31	<0.001
HbA1c, %	5.56 ± 0.69	6.23 ± 1.17	5.60 ± 1.32	6.09 ± 1.01	5.67 ± 0.90	<0.001	5.54 ± 0.68	6.10 ± 1.14	5.53 ± 0.54	6.04 ± 1.26	5.69 ± 0.87	<0.001
Fasting glucose, mmol/L	94.20 ± 17.73	115.53 ± 32.43	95.05 ± 24.17	113.47 ± 29.38	97.76 ± 22.86	<0.001	93.15 ± 17.74	110.03 ± 33.63	92.80 ± 13.19	106.65 ± 31.28	97.71 ± 24.25	<0.001

Abbreviations: HHcy, hyperhomocysteinemia; MetS, metabolic syndrome; eGFR, estimated glomerular filtration rate; TG, Triglyceride; HDL, High-density lipoprotein-cholesterol; hs-CRP, high-sensitivity c-reactive protein; WC, waist circumference; TC, total cholesterol; SBP, systolic blood pressure; DBP, diastolic blood pressure; HbA1c, hemoglobin A1c. Continuous variables are presented as means ± standard deviations and categorical variables as numbers (percentages). Normality of data was assessed using a Shapiro–Wilk test. ANOVA for continuous variables or Pearson’s chi-square test for categorical variables. Bonferroni post-hoc correction was used for multiple comparisons.

**Table 2:** Odds of CKD for the presence or absence of HHcy and MetS.

		Males			Females		
		OR	95%CI	*p*	OR	95%CI	*p*
Model 1							
	HHcy−/MetS−	Reference			Reference		
	HHcy−/MetS+	1.175	0.996–1.385	0.055	1.261	1.063–1.498	0.008
	HHcy+/MetS−	1.951	1.667–2.284	<0.001	2.323	1.563–3.453	<0.001
	HHcy+/MetS+	2.087	1.497–2.909	<0.001	4.095	2.383–7.037	<0.001
	*p*-for trend			<0.001			<0.001
Model 2							
	HHcy−/MetS−	Reference			Reference		
	HHcy−/MetS+	1.140	0.966–1.346	0.121	1.237	1.040–1.470	0.016
	HHcy+/MetS−	1.941	1.658–2.273	<0.001	2.277	1.531–3.387	<0.001
	HHcy+/MetS+	2.015	1.444–2.812	<0.001	4.006	2.329–6.892	<0.001
	*p*-for trend			<0.001			<0.001
	Age	1.008	1.003–1.013	0.002	0.991	0.984–0.997	0.005
	Smoking	0.972	0.901–1.049	0.465	0.937	0.804–1.091	0.400
	Drinking	1.078	0.981–1.186	0.120	1.012	0.891–1.149	0.858
	Physical activity	1.036	0.924–1.162	0.541	0.924	0.791–1.080	0.323
	hs-CRP	1.106	1.004–1.219	0.042	0.948	0.809–1.109	0.503
	Albumin	1.383	1.131–1.692	0.002	1.549	1.183–2.029	0.001
	HHcy+/MetS+ vs HHcy−/MetS+	1.750	1.223–2.505	0.002	3.224	1.851–5.616	<0.001
	HHcy+/MetS+ vs HHcy+/MetS−	1.070	0.746–1.535	0.712	1.847	0.942–3.622	0.074

*p*-values calculated using logistic regression. *p*-for trend was calculated using the Cochran–Armitage trend test. Abbreviations: HHcy, hyperhomocysteinemia; MetS, metabolic syndrome; CKD, chronic kidney disease; OR, odds ratio; CI, confidence interval; hs-CRP, high-sensitivity c-reactive protein. Model 1: Univariate logistic regression analysis when the age was held constant. Model 2: Multivariate logistic regression analysis adjusted for smoking, drinking, physical activity, hs-CRP level, and albumin level.

**Table 3:** Odds of a low eGFR for the presence or absence of HHcy and MetS.

		Males			Females		
		OR	95%CI	*p*	OR	95%CI	*p*
Model 1							
	HHcy−/MetS−	Reference			Reference		
	HHcy−/MetS+	0.547	0.299–1.001	0.050	0.918	0.542–1.557	0.752
	HHcy+/MetS−	4.926	3.667–6.616	<0.001	3.692	1.986–6.867	<0.001
	HHcy+/MetS+	3.814	2.034–7.153	<0.001	12.913	6.089–27.385	<0.001
	*p*-for trend			<0.001			<0.001
Model 2							
	HHcy−/MetS−	Reference			Reference		
	HHcy−/MetS+	0.540	0.295–0.989	0.046	0.925	0.544–1.574	0.775
	HHcy+/MetS−	4.923	3.664–6.615	<0.001	3.698	1.981–6.902	<0.001
	HHcy+/MetS+	3.716	1.971–7.004	<0.001	13.530	6.338–28.885	<0.001
	*p*-for trend			<0.001			<0.001
	Age	1.103	1.089–1.117	<0.001	1.139	1.116–1.163	<0.001
	Smoking	0.921	0.769–1.103	0.373	1.268	0.911–1.766	0.160
	Drinking	0.945	0.749–1.193	0.636	0.810	0.578–1.134	0.219
	Physical activity	0.950	0.721–1.250	0.712	1.226	0.819–1.837	0.322
	hs-CRP	1.014	0.676–1.523	0.945	0.863	0.451–1.650	0.655
	Albumin	1.242	0.756–2.038	0.392	1.224	0.585–2.516	0.583
	HHcy+/MetS+ vs HHcy−/MetS+	7.032	3.013–16.413	<0.001	14.637	6.090–35.179	<0.001
	HHcy+/MetS+ vs HHcy+/MetS−	0.787	0.417–1.485	0.460	2.517	1.074–5.889	0.034

*p*-values calculated using logistic regression. *p*-for trend calculated using Cochran–Armitage trend test. Abbreviations: HHcy, hyperhomocysteinemia; MetS, metabolic syndrome; OR, odds ratio; CI, confidence interval; hs-CRP, high-sensitivity c-reactive protein; eGFR, estimated glomerular filtration rate. Model 1: univariate logistic regression analysis when the age was held constant. Model 2: multivariate logistic regression analysis adjusted for smoking, drinking, physical activity, hs-CRP level, and albumin level.

**Table 4:** Odds of albuminuria for the presence or absence of HHcy and MetS.

		Males			Females		
		OR	95%CI	*p*	OR	95%CI	*p*
Model 1							
	HHcy−/MetS−	Reference			Reference		
	HHcy−/MetS+	1.238	1.046–1.466	0.013	1.273	1.064–1.523	0.008
	HHcy+/MetS−	1.568	1.318–1.866	<0.001	1.507	0.915–2.482	0.107
	HHcy+/MetS+	1.837	1.286–2.625	<0.001	3.280	1.773–6.068	<0.001
	*p*-for trend			<0.001			<0.001
Model 2							
	HHcy−/MetS−	Reference			Reference		
	HHcy−/MetS+	1.199	1.011–1.421	0.037	1.242	1.037–1.488	0.019
	HHcy+/MetS−	1.561	1.311–1.858	<0.001	1.469	0.891–2.420	0.132
	HHcy+/MetS+	1.769	1.237–2.530	0.002	3.186	1.719–5.904	<0.001
	*p*-for trend			<0.001			<0.001
	Age	0.993	0.988–0.999	0.013	0.972	0.965–0.979	<0.001
	Smoking	0.990	0.913–1.073	0.802	0.900	0.763–1.062	0.212
	Drinking	1.082	0.979–1.196	0.122	1.038	0.908–1.188	0.582
	Physical activity	1.051	0.931–1.185	0.422	0.913	0.775–1.077	0.281
	hs-CRP	1.121	1.019–1.233	0.019	0.948	0.810–1.110	0.509
	Albumin	1.399	1.132–1.728	0.002	1.662	1.250–2.209	0.000
	HHcy+/MetS+ vs HHcy−/MetS+	1.456	0.993–2.136	0.055	2.534	1.353–4.747	0.004
	HHcy+/MetS+ vs HHcy+/MetS−	1.130	0.767–1.665	0.537	2.241	1.023–4.908	0.044

*p*-values calculated using logistic regression. *p*-for trend calculated using the Cochran–Armitage trend test. Abbreviations: HHcy, hyperhomocysteinemia; MetS, metabolic syndrome; OR, odds ratio; CI, confidence interval; hs-CRP, high-sensitivity c-reactive protein. Model 1: univariate logistic regression analysis when the age was held constant. Model 2: multivariate logistic regression analysis adjusted for smoking, drinking, physical activity, hs-CRP level, and albumin level.

## Supplementary Materials

The following are available online at https://www.mdpi.com/1660-4601/17/18/6810/s1: Table S1: Exclusion criteria; Table S2: Subgroup analyses of MetS patients for calculating the odds for CKD development, low eGFR, and albuminuria development in HHcy; Table S3: Propensity score-matched logistic regression analyses of the subgroups of MetS patients for calculating the odds of CKD development, low eGFR, and albuminuria development in HHcy (*n* = 297); Table S4: Odds for CKD development, low eGFR, and albuminuria development according to individual MetS components; Table S5: Odds for CKD development for the combination of HHcy and MetS components; Table S6: Odds for low eGFR for the combination of HHcy and MetS components; Table S7: Odds of albuminuria development for the combination of HHcy and MetS components.
