# Supplementary material for: Hyperhomocysteinemia Concurrent with Metabolic Syndrome Is Independently Associated with Chronic Kidney Disease among Community-Dwelling Adults in an Urban Korean Population
Source: Int J Environ Res Public Health. 2020 Sep 18;17(18):6810. doi: 10.3390/ijerph17186810 (PMC7558526; doi:10.3390/ijerph17186810)
Supplement: Supplementary file 1 [file ijerph-17-06810-s001.pdf]

Table S1. Exclusion criteria

Exclusion criteria	ICD codes
Age < 19 years	
Incomplete data	
Menstruation on the examination day	
Pregnancy	
Pyuria and suspected urinary tract infection	
Current or past diagnosis of cancer	C00-C97 for malignant neoplasms
History of liver disease	K70 for alcoholic liver disease; K71 for toxic liver disease; K72 for hepatic failure, not elsewhere classified; K73 for chronic hepatitis, not elsewhere classified; K74 for fibrosis and cirrhosis of the liver; K75 for other inflammatory liver diseases; K76 for other diseases of the liver; and K77 for liver disorders in diseases classified elsewhere
History of ischemic heart disease	I20-I25 for ischemic heart disease
History of pulmonary disease	J30-J39 for other diseases of the upper respiratory tract, J40-J47 for chronic lower respiratory diseases, J80-J84 for other respiratory diseases principally affecting the interstitium, J85-J86 for suppurative and necrotic conditions of the lower respiratory tract, J95-J99 for other diseases of the respiratory system
History of organ transplantation	Z94
History of kidney disease	N00-N08 for glomerular diseases, N10-N16 for renal tubulo-interstitial diseases, N17-N19 for renal failure, N20-N23 for urolithiasis, N25-N29 for other disorders of the kidney and ureter, N30-N39 for other diseases of the urinary system

Abbreviations: ICD, International Statistical Classification of Diseases and Related Health Problems codes

Table S2. Subgroup analyses of MetS patients for calculating the odds for CKD development, l ow eGFR, and albuminuria development in HHcy

	Males (n=1,784)			Females (n=2,307)		
	OR	95% CI	P	OR	95% CI	P
Odds of CKD						
HHcy+ vs HHcy-	1.750	1.223-2.505	0.002	3.224	1.851-5.616	<0.001
Odds of low eGFR						
HHcy+ vs HHcy-	7.032	3.013-16.413	<0.001	14.637	6.090-35.179	<0.001
Odds of albuminuria						
HHcy+ vs HHcy-	1.456	0.993-2.136	0.055	2.534	1.353-4.747	0.004

Abbreviations: HHcy, hyperhomocysteinemia; MetS, metabolic syndrome; CKD, chronic kidney disease; eGFR, estimated glomerular filtration rate; OR, odds ratio; CI, confidence interval
P-values calculated using logistic regression.

Multivariate logistic regression analysis adjusted for age, smoking, drinking, physical activity, high-sensitivity c-reactive protein level, and albumin level.

Table S3. Propensity score-matched logistic regression analyses of the subgroups of MetS patients for calculating the odds of CKD development, low eGFR, and albuminuria development in HHcy (n=297)

	OR	95% CI	P
Odds of CKD			
HHcy+ vs HHcy-	1.509	0.989-2.303	0.056
Odds of low eGFR			
HHcy+ vs HHcy-	6.692	2.292-19.539	<0.001
Odds of albuminuria			
HHcy+ vs HHcy-	1.232	0.788-1.926	0.360

Abbreviations: HHcy, hyperhomocysteinemia; MetS, metabolic syndrome; CKD, chronic kidney disease; eGFR, estimated glomerular filtration rate; OR, odds ratio; CI, confidence interval
P-values calculated using conditional logistic regression after propensity score matching.

Table S4. Odds for CKD development, low eGFR, and albuminuria development according to individual MetS components

	Males			Females		
	OR	95% CI	P	OR	95% CI	P
Odds of CKD						
Central obesity	1.228	1.062-1.420	0.006	1.401	1.195-1.641	<0.001
High TG	1.120	0.990-1.268	0.072	1.114	0.943-1.316	0.205
Low HDL	1.005	0.838-1.206	0.957	1.248	1.066-1.461	0.006
High BP	1.044	0.927-1.175	0.480	1.184	1.011-1.387	0.036
High fasting glucose	0.991	0.874-1.123	0.884	0.993	0.837-1.177	0.932
Odds of low eGFR						
Central obesity	0.784	0.532-1.154	0.217	1.250	0.821-1.902	0.298
High TG	0.937	0.694-1.264	0.668	1.170	0.760-1.800	0.476
Low HDL	0.610	0.362-1.028	0.064	1.251	0.832-1.880	0.282
High BP	0.890	0.670-1.183	0.424	0.996	0.653-1.518	0.984
High fasting glucose	0.837	0.615-1.139	0.258	0.704	0.439-1.128	0.145
Odds of albuminuria						
Central obesity	1.308	1.125-1.520	<0.001	1.415	1.197-1.673	<0.001
High TG	1.164	1.022-1.326	0.022	1.101	0.923-1.314	0.284
Low HDL	1.076	0.891-1.298	0.447	1.270	1.075-1.500	0.005
High BP	1.045	0.923-1.184	0.486	1.210	1.024-1.429	0.025
High fasting glucose	1.012	0.887-1.154	0.857	1.056	0.884-1.263	0.547
Central obesity	1.308	1.125-1.520	<0.001	1.415	1.197-1.673	<0.001

Abbreviations: MetS, metabolic syndrome; CKD, chronic kidney disease; eGFR, estimated glomerular filtration rate; TG, triglyceride; HDL, high-density lipoprotein-cholesterol; BP, blood pressure; OR, odds ratio; CI, confidence interval
P-values calculated using logistic regression.
Multivariate logistic regression analysis adjusted for age, smoking, drinking, physical activity, high-sensitivity c-reactive protein level, and albumin level.

Table S5. Odds for CKD development for the combination of HHcy and MetS components

	Males			Females		
	OR	95% CI	P	OR	95% CI	P
Central obesity						
HHcy+/Central obesity+ vs HHcy-/Central obesity-	2.318	1.691-3.177	<0.001	4.319	2.681-6.958	<0.001
HHcy+/Central obesity+ vs HHcy-/Central obesity+	1.255	0.947-1.662	0.114	1.460	1.032-2.067	0.033
HHcy+/Central obesity+ vs HHcy+/Central obesity-	1.221	0.866-1.724	0.255	2.322	1.205-4.472	0.012
High TG						
HHcy+/High TG+ vs HHcy-/High TG-	2.132	1.656-2.744	<0.001	2.242	1.228-4.093	0.009
HHcy+/High TG+ vs HHcy-/High TG+	0.987	0.694-1.402	0.940	0.968	0.711-1.319	0.838
HHcy+/High TG+ vs HHcy+/High TG-	1.121	0.840-1.497	0.438	0.786	0.387-1.596	0.505
Low HDL						
HHcy+/Low HDL+ vs HHcy-/Low HDL-	1.803	1.141-2.848	0.012	2.242	1.228-4.093	0.009
HHcy+/Low HDL+ vs HHcy-/Low HDL+	0.879	0.656-1.178	0.388	1.058	0.752-1.488	0.746
HHcy+/Low HDL+ vs HHcy+/Low HDL-	0.882	0.551-1.412	0.602	1.453	0.752-2.808	0.266
High BP						
HHcy+/High BP+ vs HHcy-/High BP-	2.314	1.738-3.083	<0.001	2.254	1.115-4.558	0.024
HHcy+/High BP+ vs HHcy-/High BP+	0.937	0.676-1.299	0.697	0.913	0.670-1.244	0.564
HHcy+/High BP+ vs HHcy+/High BP-	1.214	0.928-1.588	0.158	0.947	0.488-1.838	0.872
High fasting glucose						
HHcy+/High fasting glucose+ vs HHcy-/High fasting glucose-	2.079	1.529-2.827	<0.001	2.605	1.209-5.615	0.015
HHcy+/High fasting glucose+ vs HHcy-/High fasting glucose+	0.958	0.695-1.319	0.790	0.697	0.529-0.919	0.011
HHcy+/High fasting glucose+ vs HHcy+/High fasting glucose-	1.014	0.757-1.357	0.928	1.280	0.641-2.555	0.484

Abbreviations: HHcy, hyperhomocysteinemia; MetS, metabolic syndrome; CKD, chronic kidney disease; TG, triglyceride; HDL, high-density lipoprotein-cholesterol; BP, blood pressure; OR, odds ratio; CI, confidence interval;

P-values calculated using logistic regression.

Multivariate logistic regression analysis adjusted for age, smoking, drinking, physical activity, high-sensitivity c-reactive protein level, and albumin level.

Table S6. Odds for low eGFR for the combination of HHcy and MetS components

	Males			Females		
	OR	95% CI	P	OR	95% CI	P
Central obesity						
HHcy+/Central obesity+ vs HHcy-/Central obesity-	5.257	3.032-9.115	<0.001	12.743	6.231-26.061	<0.001
HHcy+/Central obesity+ vs HHcy-/Central obesity+	1.334	0.560-3.177	0.515	1.378	0.568-3.342	0.479
HHcy+/Central obesity+ vs HHcy+/Central obesity-	1.212	0.695-2.115	0.497	2.565	1.092-6.023	0.031
High TG						
HHcy+/High TG+ vs HHcy-/High TG-	4.862	3.048-7.758	<0.001	6.221	2.643-14.644	<0.001
HHcy+/High TG+ vs HHcy-/High TG+	0.551	0.220-1.380	0.203	1.411	0.578-3.446	0.450
HHcy+/High TG+ vs HHcy+/High TG-	0.954	0.594-1.531	0.845	0.910	0.368-2.253	0.838
Low HDL						
HHcy+/Low HDL+ vs HHcy-/Low HDL-	4.445	1.977-9.996	<0.001	9.384	3.858-22.824	<0.001
HHcy+/Low HDL+ vs HHcy-/Low HDL+	0.940	0.398-2.219	0.887	2.292	0.758-6.926	0.142
HHcy+/Low HDL+ vs HHcy+/Low HDL-	0.840	0.382-1.846	0.665	2.683	1.134-6.348	0.025
High BP						
HHcy+/High BP+ vs HHcy-/High BP-	4.297	2.498-7.390	<0.001	6.788	2.500-18.436	<0.001
HHcy+/High BP+ vs HHcy-/High BP+	1.625	0.538-4.914	0.389	0.913	0.670-1.244	0.564
HHcy+/High BP+ vs HHcy+/High BP-	0.843	0.542-1.313	0.450	0.903	0.370-2.201	0.822
High fasting glucose						
HHcy+/High fasting glucose+ vs HHcy-/High fasting glucose-	4.715	2.688-8.271	<0.001	6.427	2.128-19.406	<0.001
HHcy+/High fasting glucose+ vs HHcy-/High fasting glucose+	0.591	0.252-1.382	0.225	0317	0.147-0.684	0.003
HHcy+/High fasting glucose+ vs HHcy+/High fasting glucose-	1.070	0.669-1.710	0.778	1.110	0.446-2.765	0.822

Abbreviations: HHcy, hyperhomocysteinemia; MetS, metabolic syndrome; eGFR, estimated glomerular filtration rate; TG, triglyceride; HDL, high-density lipoprotein-cholesterol; BP, blood pressure; OR, odds ratio; CI, confidence interval;

P-values calculated using logistic regression.

Multivariate logistic regression analysis adjusted for age, smoking, drinking, physical activity, high-sensitivity c-reactive protein level, and albumin level.

Table S7. Odds of albuminuria development for the combination of HHcy and MetS components

	Males			Females		
	OR	95% CI	P	OR	95% CI	P
Central obesity						
HHcy+/Central obesity+ vs HHcy-/Central obesity-	2.002	1.425-2.812	<0.001	3.276	1.902-5.643	<0.001
HHcy+/Central obesity+ vs HHcy-/Central obesity+	1.201	0.899-1.604	0.215	1.428	0.992-2.056	0.055
HHcy+/Central obesity+ vs HHcy+/Central obesity-	1.261	0.870-1.828	0.222	2.628	1.208-5.718	0.015
High TG						
HHcy+/High TG+ vs HHcy-/High TG-	1.858	1.415-2.440	<0.001	1.725	0.850-3.498	0.131
HHcy+/High TG+ vs HHcy-/High TG+	1.089	0.751-1.578	0.654	0.896	0.650-1.236	0.503
HHcy+/High TG+ vs HHcy+/High TG-	1.214	0.886-1.662	0.227	0.921	0.401-2.112	0.845
Low HDL						
HHcy+/Low HDL+ vs HHcy-/Low HDL-	1.637	1.002-2.675	0.049	2.065	0.983-4.338	0.055
HHcy+/Low HDL+ vs HHcy-/Low HDL+	0.879	0.656-1.178	0.388	1.058	0.752-1.488	0.746
HHcy+/Low HDL+ vs HHcy+/Low HDL-	0.954	0.576-1.580	0.856	1.441	0.661-3.143	0.359
High BP						
HHcy+/High BP+ vs HHcy-/High BP-	2.073	1.523-2.821	<0.001	1.972	0.873-4.454	0.103
HHcy+/High BP+ vs HHcy-/High BP+	0.911	0.651-1.273	0.584	0.913	0.670-1.244	0.564
HHcy+/High BP+ vs HHcy+/High BP-	1.295	0.965-1.739	0.085	1.201	0.554-2.606	0.643
High fasting glucose						
HHcy+/High fasting glucose+ vs HHcy-/High fasting glucose-	1.748	1.249-2.447	<0.001	2.422	1.001-5.857	<0.001
HHcy+/High fasting glucose+ vs HHcy-/High fasting glucose+	0.998	0.715-1.394	0.993	0.806	0.602-1.079	0.147
HHcy+/High fasting glucose+ vs HHcy+/High fasting glucose-	0.926	0.670-1.281	0.644	1.623	0.729-3.613	0.236

Abbreviations: HHcy, hyperhomocysteinemia; MetS, metabolic syndromeTG, triglyceride; HDL, high-density lipoprotein-cholesterol; BP, blood pressure; OR, odds ratio; CI, confidence interval;
P-values calculated using logistic regression.
Multivariate logistic regression analysis adjusted for age, smoking, drinking, physical activity, high-sensitivity c-reactive protein level, and albumin level.
